# Non-utilization Is Not the Best Way to Manage Lowland Meadows in Hulun Buir

**DOI:** 10.3389/fpls.2021.704511

**Published:** 2021-07-16

**Authors:** Guoxu Ji, Bing Li, Hang Yin, Guofu Liu, Yuying Yuan, Guowen Cui

**Affiliations:** ^1^Department of Grassland Science, College of Animal Science and Technology, Northeast Agricultural University, Harbin, China; ^2^Institute of Environment and Sustainable Development in Agriculture, Chinese Academy of Agricultural Sciences, Beijing, China

**Keywords:** utilization mode, plant community, soil physicochemical properties, temperature and precipitation, *Carex meyeriana*

## Abstract

*Carex meyeriana* lowland meadow is an important component of natural grasslands in Hulun Buir. However, in Hulun Buir, fewer studies have been conducted on *C. meyeriana* lowland meadows than on other grassland types. To determine the most appropriate utilization mode for *C. meyeriana* lowland meadows, an experiment was conducted in Zhalantun city, Hulun Buir. Unused, moderately grazed, heavily grazed and mowed meadow sites were selected as the research objects. The analysis of experimental data from 4 consecutive years showed that relative to the other utilization modes, mowing and moderate grazing significantly increased *C. meyeriana* biomass. Compared with non-utilization, the other three utilization modes resulted in a higher plant diversity, and the moderately grazed meadow had the highest plant community stability. Moreover, principal component analysis (PCA) showed that among the meadow sites, the mowed meadow had the most stable plant community and soil physicochemical properties. Structural equation modeling (SEM) showed that grazing pressure was less than 0.25 hm^2^/sheep unit and that plant biomass in *C. meyeriana* lowland meadow increases with increasing grazing intensity, temperature and precipitation.

## Introduction

Vegetation is the most basic component of terrestrial ecosystems and interacts with temperature, precipitation, topography, and other factors to determine the ecosystem type (Ge et al., 2004; Liu and Tian, 2010). Vegetation is also the most easily changed component of ecosystems. Changes in the utilization pattern or intensity of vegetation, such as changes in grazing or mowing practices, can directly affect plant species presence and diversity and thus the stability of an entire grassland ecosystem (Socher et al., 2012; Li et al., 2018b). A series of experimental studies have been carried out to understand how external disturbances affect grassland plant communities and how to protect and utilize grasslands.

Overgrazing is one of the main causes of grassland degradation in China and even worldwide (Wang et al., 2011). The impacts of grazing on grassland have consistently been a focus of research. At present, there are two major hypotheses regarding the impact of grazing on grassland plant diversity: the grazing optimization hypothesis and the intermediate disturbance hypothesis. The latter has been supported by a large number of experiments (Fensham et al., 2014; Beck et al., 2015; Qin et al., 2019), although some experiments provide no support, in some cases contradicting this hypothesis (Rutherford and Powrie, 2013; Ren et al., 2018). These differences among experimental results indicate that the effects of grazing on grassland communities are complex and result from the combined actions of multiple factors; a change in any one factor, such as livestock species, grass type, a climatic conditions or a soil physicochemical property, can greatly affect the experimental results (Liu et al., 2015; Chu et al., 2019; Ji et al., 2020). Compared with the effect of grazing, the effect of mowing on grassland is more spatially homogeneous and can directly lead to significant reductions in the height and cover of grassland plant communities as well as a reduction in the accumulation of grassland litter (Kose et al., 2019). Current opinions on the impact of mowing on grassland plant diversity are more consistent and include the view that moderate mowing cannot only help stabilize grassland plant communities but also promote grassland plant compensatory growth (Yang et al., 2012). Experiments conducted by Bernhardt-Römermann et al. (2011), Socher et al. (2012), and Nakahama et al. (2016) demonstrated that moderate mowing was beneficial for maintaining or increasing the stability of plant community diversity and productivity. In grassland ecosystems, the soil and vegetation interact with each other; changes in plant communities affect the use of soil nutrients and ultimately change the soil chemical properties, and changes in soil chemical properties affect plant communities.

Numerous experiments have shown that grazing (Teague et al., 2011; Zhang et al., 2019), mowing (Vermeire et al., 2020; Wang et al., 2020a), and climate change (Hoffmann et al., 2016; Hu et al., 2018) can directly or indirectly affect soil physicochemical properties. Grazing or mowing increase the soil bulk density (SBD) (though not always significantly) until it reaches a stable level (Okach et al., 2018; Xu et al., 2018; Batista et al., 2019). However, the impact of grazing or mowing on soil chemical properties do not exhibit consistent trends (Luo et al., 2019; Yang et al., 2020). Some scholars have found that grazing or mowing does not significantly change soil chemical properties (Kitchen et al., 2009; Swacha et al., 2018; Nakano et al., 2020). However, many experiments have proven that grazing increases soil organic carbon (Wei et al., 2011) or resulted in soil nutrient loss (Zhan et al., 2020).

Therefore, although many experiments have been carried out to study the effects of various utilization modes on grassland, further research is warranted because of the differing effects of experimental factors, such as treatment methods and grassland types. Moreover, global climate change has become an indisputable fact (Ciais et al., 2014), and the responses of grasslands, which are highly sensitive, to climate change are complex (Polley et al., 2014; Ganjurjav et al., 2016; Salick et al., 2019; Harrison, 2020). Therefore, continued exploration of the most appropriate uses of various grassland types under climate change conditions is necessary. The Hulun Buir grassland, located at the eastern edge of the Eurasian grassland, is an important component of the grasslands in northern China and forms an ecological barrier. Many experiments regarding the utilization status of the Hulun Buir grassland have been carried out, but few have been carried out in *Carex meyeriana* lowland meadow. *C. meyeriana* is one of the most important grassland constructive species and a dominant species in the lowland meadows of the Hulun Buir grassland; accordingly, *C. meyeriana* lowland meadow is one of the most important grassland types in this region. Therefore, it is worth discussing whether the intermediate disturbance hypothesis is supported in the utilization of *C. meyeriana* lowland meadow. In this study, *C. meyeriana* lowland meadow sites under four grassland utilization modes (non-use, mowing, moderate grazing, and heavy grazing) were selected as the research objects. The species composition, biomass, plant diversity, and soil physicochemical properties of these meadow sites were studied to analyze the difference in the impact of four utilization patterns on *C. meyeriana* lowland meadows, and to explore the rational utilization of *C. meyeriana* lowland meadows.

## Materials and Methods

### Study Site and Experimental Design

The experimental sites were located in Zhalantun city, Hulun Buir, Inner Mongolia, which is a typical temperate zone characterized by a semi-arid continental monsoon climate. The mean annual temperature is 3.7°C, and the monthly mean temperature ranges from 23.8 to −17.1°C (January to July). The annual mean precipitation is 565.9 mm, and 77.5% of the precipitation occurs from June to September (data from Zhalantun Meteorological Station, China Meteorological Data Network, 2015).

The experiment was carried out in 2015–2018. Plant community characteristics and soil physicochemical properties in the *C. meyeriana* lowland meadow were studied under four utilization modes: no utilization (UN), mowing once a year (MO), moderate grazing (MG), and heavy grazing (HG). The mowing site was mowed once every year in mid-late July (during a sunny and rainless period) to a stubble height of 3 cm. Based on the grass yield of the natural grassland in the study area, the appropriate stocking capacity was approximately 0.4 hm^2^/sheep unit. Therefore, after calculating the actual stocking capacity of the two grazing sites, we determined the grazing intensity of the two sites: approximately 0.37 hm^2^/sheep unit for the MG site and approximately 0.25 hm^2^/sheep unit for the HG site. Healthy sheep of the same variety (Mongolian sheep, one of the major local breeds) and of similar weight and age were selected as grazing livestock. The grazing time was from May to October each year, with no grazing occurring in the sites outside of this time. Both grazing sites were open-grazed, without fencing. During the experimental period, the grazing intensity at each of the two grazed sites remained stable. Each site was divided into three plots of similar vegetation status. The area of each experimental site was at least 1 × 10^4^ m^2^.

All four sites were in *C. meyeriana* lowland meadow, and the soil is meadow soil (Chinese classification), corresponding to Ustolls and Mollisols in the US Soil Taxonomy classification. Before the experiment was conducted, all four sites were stable in their use patterns and did not suffer any human disturbance or damage other than the use pattern. Basic information on the plots and the climate data during the experimental period is provided in Tables 1, 2, respectively.

**TABLE 1:** Basic information on the test sites.

Plot	Longitude	Latitude	Altitude	Grassland type	Grassland type	Soil type	Land use
UN	121°33′42.33″	47°35′52.45″	678 m	Lowland meadow	*C. meyeriana*	Meadow soil	Unused
MO	121°50′28.59″	47°40′05.31″	515 m	Lowland meadow	*C. meyeriana*	Meadow soil	Mowing once a year
MG	121°35′59.04″	47°37′14.50″	705 m	Lowland meadow	*C. meyeriana*	Meadow soil	Moderate grazing
HG	122°01′11.68″	47°37′29.81″	427 m	Lowland meadow	*C. meyeriana*	Meadow soil	High grazing

**TABLE 2:** Temperature and precipitation data during the test period.

Item	2015	2016	2017	2018
Annual precipitation (mm)	696.60	560.90	319.40	584.10
Annual average daily temperature (°C)	4.60	3.76	4.40	3.93
Average daily temperature from January to July (°C)	3.93	1.59	3.12	0.03
Rainfall from January to July (mm)	301.40	275.10	96.40	127.10
Average daily temperature during the growing season (°C)	17.31	16.87	17.70	16.64
Rainfall during the growing season (mm)	278.00	252.00	74.40	100.70
Rainfall from November of the previous year to the next April (mm)	35.90	34.40	43.80	41.00

### Plant Measurements and Soil Sampling

To eliminate the impact of marginal effects on the grassland plant communities, at the beginning of July in each year (usually approximately July 10th), three main quadrats (1 × 1 m) that were at least 10 m from the boundary of each plot and at least 30 m from each other were randomly selected in each sampling plot. Four additional quadrats were selected within 2–5 m of each main quadrat in the four cardinal directions. All plant species in each quadrat were recorded, and the height, number and absolute coverage of each species were measured. Then, the above-ground parts of the plants were clipped for the determination of above-ground biomass.

Soil samples were collected from two soil layers (0–20 and 21–40 cm) in each quadrat. Five soil cores (35-mm diameter) were randomly collected in each quadrat, and the soil samples were air-dried before the analysis of their physicochemical properties.

### Calculation of Plant Community Characteristics and Determination of Soil Physicochemical Properties

The mowed plants were dried in an oven at 105°C for 15 min and then dried at 65°C for 24–48 h to obtain the above-ground dry weight of the plants in each plot. Important values of *C. meyeriana* and other grasses (Zhao et al., 2013) were calculated, and plant diversity indices, including species richness, the Simpson, Shannon-Wiener, and Pielou indices (Smith and Wilson, 1996; Wang et al., 2019), and community stability (Liu et al., 2020) were calculated. The indices were calculated with the following formulas:

(1) $$Speciesrichnessindex:Ds=\frac{S-1}{\ln N}$$

(2) $$Simpsonindex:D=1-\left[\sum_{i=1}^{s}{\left(P_{i}\right)}^{2}\right]$$

(3) $$Shannon-Wienerindex:H=-\sum_{i=1}^{s}\left(P_{i}\ln P_{i}\right)$$

(4) $$Pielouindex:E=\frac{H}{\ln S}$$

where S is the total number of species occurring within the plot; $P_{i}=\frac{N_{i}}{N}$, N is the total number of individuals in the site, and Ni is the number of species I.

The soil water content (SWC) of each soil sample was measured after drying at 105°C for 48 h. The SBD was calculated as the mass of the oven-dried soil (105°C) divided by the core volume. The soil pH was determined from a 1:2 (soil/water ratio) slurry of oven-dried soil and deionized water, which was measured with a pH meter after incubation at 25°C for 30 min. The soil total nitrogen (STN) content was measured using the Kjeldahl method (Kalembasa and Jenkinson, 1973). STN concentration was measured using the Bremner (1965). Soil available nitrogen (AN), available potassium (AK), and available phosphorous (AP) contents were measured as described previously (Wu et al., 2011).

### Data Analysis

All data are expressed as the mean ± standard error (SE). Samples were subjected to ANOVA to distinguish significant differences by SAS software (version 9.2, SAS Institute, United States). The LSDs of the values were decided at the level of significance (α = 0.05). Stepwise regression analysis was performed with SAS. Principal component analysis (PCA) was performed using R (version 3.6.3, packages: “FactoMineR” and “factoextra”). Structural equation modeling (SEM) was performed with Amos 24.0 (SPSS Statistics 24.0).

## Results

### Effects of Utilization Mode on the Plant Community Characteristics and Soil Physicochemical Properties of Lowland Meadow

**FIGURE 1:**
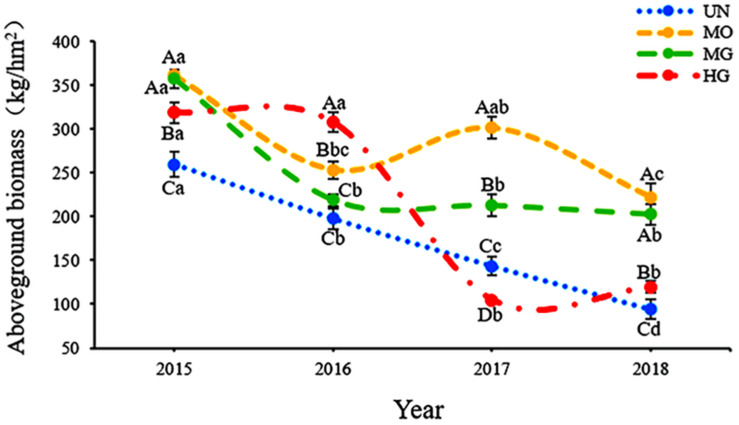
Interannual variation in grassland plant biomass under different utilization patterns. Different capital letters indicate significant differences in plant biomass among sites in the same year at the 0.05 level, and different lowercase letters indicate significant differences in plant biomass between years within a site.

Under the different utilization modes, above-ground plant biomass in the four grassland sites exhibited different trends. As shown in Figure 1, the biomass at the UN site decreased each year, the biomass at the MO and HG sites fluctuated, and the biomass at the MG site stabilized after 2016; its coefficient of variation was only 7.55%.

**FIGURE 2:**
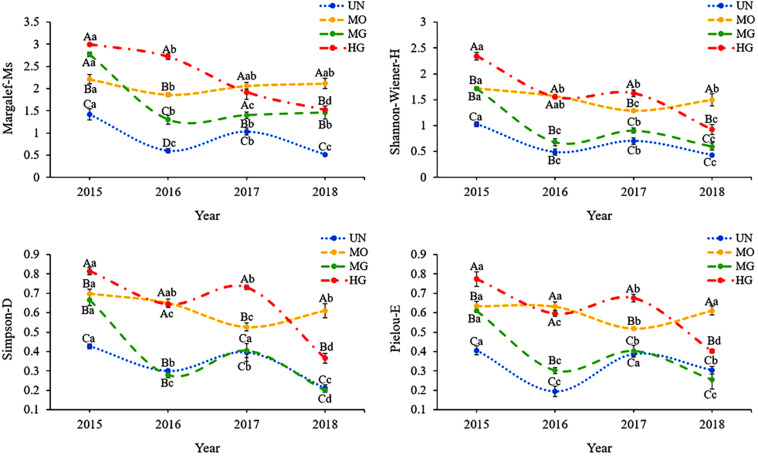
Interannual variation in grassland plant diversity under different utilization patterns. Different capital letters indicate significant differences in a plant diversity index among sites in the same year at the 0.05 level, and different lowercase letters indicate significant differences in a plant diversity index between years within a site.

Figure 2 shows that plant diversity similarly exhibited different patterns among the four sites. Among the sites, the UN site had the lowest plant diversity, which was significantly lower than that at the MO and HG sites (*p* < 0.05). Plant diversity at the UN site showed a decreasing, increasing, and then decreasing trend, and the plant diversity at the MG site exhibited a similar pattern. Starting in 2016, plant diversity was very similar between these two sites; among the indices, only species richness differed, being significantly higher at the MG site than at the UN site (*p* < 0.05). In addition, among the sites, the MO site exhibited the most stable plant diversity, which fluctuated greatly only in 2017. The variation coefficient of each of the four diversity indices did not exceed 10.11%. Plant abundance at the HG site decreased each year, and the values of the other three diversity indices fluctuated greatly at this site, with the minimum coefficient of variation being 22.31%.

To calculate community stability at the four sites, the distance from the stable point in each community was calculated via the distance formula (Supplementary Table 1), and stability exhibited the order UN (70.90) > MO (70.80) > HG (70.78) > MG (70.77).

**FIGURE 3:**
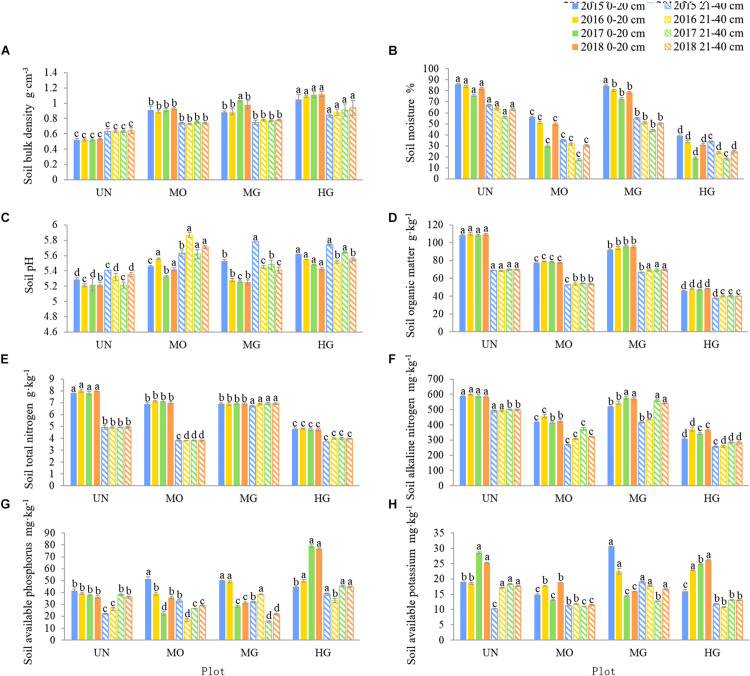
Interannual variation in soil physicochemical properties under different utilization patterns. Different letters indicate significant differences in a physicochemical property of the same soil layer among sites within a year at the 0.05 level.

Figure 3 shows that compared with UN, MO, and HG significantly increased SBD and soil pH while significantly reducing the SMC, SOM, STN, and alkali-hydrolysable nitrogen contents. In addition, compared with UN, MG also significantly increased SBD and pH, significantly reduced SOM contents at 0–20 cm, and led to the convergence of the STN and alkali-hydrolysable nitrogen contents in 0–40 cm soil. However, there was no uniform change trend for AP or AK. In addition, the SBD and the SOM and STN contents were very stable under all four utilization modes.

**FIGURE 4:**
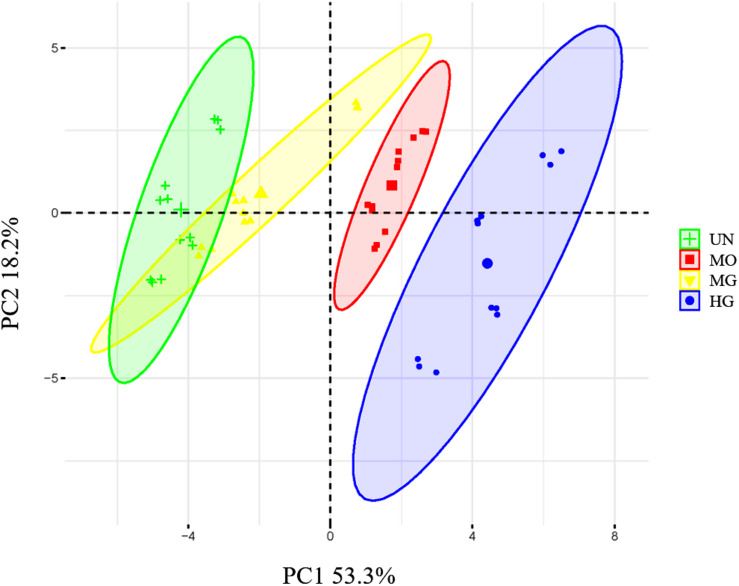
Principal component analysis (PCA) of grassland plant community characteristics and soil physicochemical properties.

A PCA of the characteristics of the plant communities and the soil physicochemical properties was conducted. The properties of plants and soil were most similar between the MG and UN sites and most stable at the MO site. Among the modes, HG changed the plant community and soil physicochemical properties of the *C. meyeriana* lowland meadow most significantly (Figure 4).

### Analysis of Key Factors Affecting Plant Biomass Under Different Utilization Modes

To clarify the key factors affecting the above-ground biomass of grassland plants under different utilization modes, stepwise regression analysis of the characteristics of grassland plant communities, soil physicochemical properties, and hydrothermal data from January to July and hydrothermal data from the growth period was performed. Table 3 shows that the key factors affecting the plant biomass of *C. meyeriana* meadow were consistent between the MG and HG sites and not significantly related to the hydrothermal conditions. However, the key factors that affected plant biomass significantly differed between the UN and MO sites because of the differing hydrothermal conditions. In all four sites, there was a significant positive correlation between plant biomass and the temperature from January to July.

**TABLE 3:** Key factors determining plant biomass under different utilization modes.

Plot	Stepwise regression model	Significance
UN	*PB* = 31.795 × AN1-42.195 × TN2-3.384 × SI-849.491	AN1 *p <* 0.001, TN2 *p <* 0.001, SI *p <* 0.001
	(*R*^2^ = 1.000, *p <* 0.001)	
	*PB* = 32.229 × AP1+0.121 × AN2-6.304 × SI-1131.540	AP1 *p <* 0.001, AN2 *p <* 0.001, SI *p <* 0.001
	(*R*^2^ = 1.000, *p <* 0.001)	
MO	*PB* = 31.383 × T-158.064 × TN1+1326.581	T *p <* 0.001, TN1 *p <* 0.001
	(*R*^2^ = 0.999, *p <* 0.001)	
	*PB* = 2988.246-539.983 × WI-46.132 × SOM2	WI *p <* 0.001, SOM2 *p <* 0.001
	(*R*^2^ = 0.996, *p <* 0.001)	
MG	*PB* = 438.154 × pH1+94.379 × pH2-2.217 × SI-2606.995	pH1 *p <* 0.001, pH2 *p <* 0.001, SI *p <* 0.001
	(*R*^2^ = 1.000, *p <* 0.001)	
	*PB* = 438.154 × pH1+94.379 × pH2-2.217 × SI-2606.995	pH1 *p <* 0.001, pH2 *p <* 0.001, SI *p <* 0.001
	(*R*^2^ = 1.000, *p <* 0.001)	
HG	*PB* = 2.176 × AP1+0.838 × SOM210.697 × AN2+2936.966	AP1 *p <* 0.001, SOM2 *p <* 0.001, AN2 *p <* 0.001
	(*R*^2^ = 1.000, *p <* 0.001)	
	*PB* = 2.176 × AP1+0.838 × SOM2-10.697 × AN2+2936.966	AP1 *p <* 0.001, SOM2 *p <* 0.001, AN2 *p <* 0.001
	(*R*^2^ = 1.000, *p <* 0.001)	

*PB, plant biomass, AN1, 0–20 cm soil alkaline hydrolysis nitrogen; AN2, 21–40 cm soil alkaline hydrolysis nitrogen; TN1, 0–20 cm soil total nitrogen; TN2, 21–40 cm soil total nitrogen; pH1, 0–20 cm soil pH; pH2, 21–40 cm soil pH; AP1, 0–20 cm soil available phosphorus; SOM2, 21–40 cm soil organic matter; SI, Shannon Index; WI, importance value of weeds; T, temperature from January to July. The first stepwise regression equation was constructed based on the hydrothermal data from January to July, and the second stepwise regression equation was based on the hydrothermal data during the plant growth period.*

**FIGURE 5:**
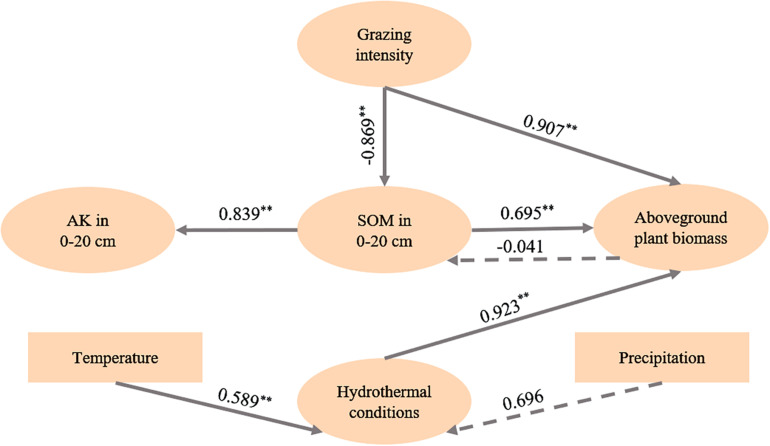
Effects of grazing intensity and hydrothermal conditions on plant biomass and soil chemical properties. ** indicates *p <* 0.001. Solid lines indicate that the correlation is significant; dotted lines indicate that the correlation is not significant. Hydrothermal conditions refer to the precipitation and average daily temperature from January to July. Structural equation model fitting indices: χ^2^/df = 1.070, RMSEA = 0.086, CFI = 0.997, NFI = 1.000, NNFI = 0.985, SRMR = 0.083.

To clarify the interactions among grazing intensity, hydrothermal conditions, soil physicochemical properties and plant biomass under grazing conditions, we performed SEM and obtained the structural equation model shown in Figure 5. Figure 5 shows that an increase in grazing intensity can be expected to increase the biomass of above-ground plants and decrease the SOM content in the 0–20 cm layer. In addition, the increase in temperature also contributed to the increase in plant biomass.

## Discussion

### Effects of Utilization Mode on Grassland Plant Communities

Some scholars have demonstrated that external disturbances can affect the biomass of grassland plants (Kawada et al., 2008; Koyama et al., 2016). Our results showed that MO, MG, and HG all significantly increased plant biomass in the *C. meyeriana* lowland meadows (although HG reduced plant biomass in 2017, probably due to the little precipitation in that year). This result differs from that of Wang and Wesche (2016) and Lu et al. (2017). This difference is due to the simple community composition of the *C. meyeriana* meadow in its native state and the absolute dominance of *C. meyeriana* in the community. Even though the precipitation decreased in 2017 and the percentage of some drought-loving plants increased, *C. meyeriana* still accounted for 50.09% of the community (Supplementary Table 1). However, compared to that of other types of grasses, the quality of *C. meyeriana* was lower. Therefore, after MO and MG, the proportion of miscellaneous grasses in *C. meyeriana* meadow increased, resulting in increases in plant biomass. Although HG increased plant biomass, meadow biomass decreased each year with increasing number grazing years under HG, especially in years with reduced precipitation. This increase under HG may reflect plant compensatory growth (McNaughton, 1979; Sun et al., 2019). In addition, the results indicated that the effect of grazing on grassland biomass is complex (Jinhua et al., 2010; Li et al., 2019).

In this study, the different utilization modes had different impacts on plant diversity. Among the sites, the MO site exhibited the least variation in the four diversity indices, and the interannual variation in the number of plant species was also minimal at this site. These findings indicate that MO helped maintain plant diversity in the lowland meadow. These results were obtained because mowing has relatively homogeneous effects on grasslands, prevents competing dominant species from eliminating competing vulnerable species, improves canopy radiation, ensures the growth of various plant species and thus maintains species diversity (Yang et al., 2012; Kose et al., 2019). Our results are also consistent with the research results of many studies (Verrier and Kirkpatrick, 2005; Socher et al., 2013; Smith et al., 2018). The mowing-associated factors that affect the diversity of grasses include mowing time, stubble height and mowing frequency (Malin et al., 2018; Chen et al., 2021). In addition, grassland type and climate conditions can affect experimental results (Su et al., 2017). For these reasons, the results of this experiment partially differed from the findings of Socher et al. (2012) and Lepš (2014). In the present experiment, the impacts of mowing and grazing on the diversity of *C. meyeriana* lowland meadows were the focus, and the impacts of specific mowing practices on plant diversity were not studied in depth. The results of this study show only that successive mowing maintained the stability of the *C. meyeriana* meadow plant community and do not reveal whether mowing once per year was the best management method or whether this mowing frequency was the optimal frequency for this management model.

In this study, the plant diversity indices at the HG site were all higher than those at the MG site, indicating that an increase in grazing intensity could lead to increases in the number of plant species and plant diversity in lowland meadows. The abundance of weeds at the HG site was higher than that at the MG site, which explained why plant diversity was higher at the HG site (Supplementary Table 2). This result is similar to the results of an experiment conducted by Zhang et al. (2017) in the Horqin Grassland, where the dominance of annual and other weeds increased under grazing conditions. In addition, the results of community stability calculations also indicated that the community composition at the MG site was more stable than that at the HG site (A lower score indicates a more stable community; therefore, the MG plant community had the highest stability, Supplementary Table 1). In contrast, Spellmeier et al. (2019) in swamps of southwestern Brazil showed that grazing reduced plant species richness. Spellmeier et al. (2019) selected a test site with naturally high plant richness that was in its native state; the data showed that the species count at that site under non-degraded conditions was much higher than that in the *C. meyeriana* meadow considered in this study. In conclusion, our experimental results indicated that UN (enclosure) is not the most appropriate management method for meadows in the long run (Sun et al., 2020).

### Effects of Utilization Mode on Grassland Soil Physicochemical Properties

As with plant diversity, the soil physicochemical properties were affected differently by the different utilization patterns. The results showed that compared with UN, MO, MG, and HG all led to increases in SBD and pH, although to different degrees, and that both properties tended to stabilize once the utilization mode remained unchanged for a long time. The reasons for the increases in pH may be the decrease in deadfall due to mowing or grazing, greater evaporation of soil water and increased salt accumulation, and the decomposition of livestock excrement, which increase soil pH (Gao et al., 2014; Hao and He, 2019; Cai et al., 2020). In contrast, SWC decreased to different degrees under the different utilization modes and was higher in the shallow soil layer than in the deep soil layer, especially at the MO and HG sites; this result may have been due to the increased compactness and decreased permeability of the soil, which would inhibit water entry into the deep soil layer (Ran et al., 2019). Furthermore, the different utilization modes led to different degrees of reduction in the SOM, STN and alkali-hydrolysable nitrogen contents. Mowing or grazing significantly reduces litter formation, thereby greatly reducing the possibility for external nutrient inputs into the soil; furthermore, some utilization modes can accelerate the consumption of soil nutrients by grassland plants and lead to a serious imbalance in soil nutrients (Wang et al., 2010). However, the effects on the soil AP and AK contents were not the same among the different utilization modes, which is consistent with the results of Lu et al. (2015). In summary, different utilization modes have different effects on soil chemical properties in different grassland types and at different utilization intensities.

Vegetation and soil are the most fundamental and important components of grassland ecosystems. Changes in soil and vegetation affect other properties of vegetation and soil and grassland ecosystems (Zuo et al., 2018). Furthermore, the various utilization modes differ in the type and intensity of influences they have on plants and soil (Sun et al., 2017; Guangyu et al., 2018; Wang et al., 2020b), for example, mowing directly and rapidly changes the structure and biomass of the plant community, but the changes in soil physical and chemical properties require some time to accumulate (Zong and Shi, 2020). In comparison to those of mowing, the impacts of HG on grassland plants and soil are more direct (Zhang et al., 2018a, b). In this study, MO did not strengthen the correlation between biomass and soil nutrient levels, whereas MG and HG strengthened the correlation between biomass and STN. Furthermore, the correlations between plant community diversity and soil physicochemical properties differed among the four sites (Supplementary Tables 3A,B). This finding is similar to the results of many studies in which the correlations between plant communities and soil physicochemical properties were not uniform across different grassland types and utilization patterns (Herrero-Jáuregui and Oesterheld, 2017; Fayiah et al., 2019; Gebregergs et al., 2019).

### Analysis of Factors Affecting Grassland Plant Biomass Under Grazing

In recent years, extreme droughts and urban flooding events caused by global climate change have occurred frequently (Zheng et al., 2012; Zhou et al., 2019). According to statistical records, until 2010, the average annual precipitation in the study area was 486.4 mm, and the average annual temperature was 3.18°C (Tsolgor., 2015). During the experimental period, from 2015 to 2018, the annual rainfall in 2017 was much lower than 486.4 mm, but the annual precipitation in the other 3 years was above the average (Table 2). Although they fluctuated greatly, the temperatures in each year were all above the historical average. The local hydrothermal changes in the study area represent a microcosm of global climate change; that is, temperature increases and extreme precipitation have become frequent. Therefore, to manage natural grasslands more scientifically, it would be helpful to study the influence of hydrothermal change on grassland ecosystems.

Existing studies have shown that climate change has significantly affected the above-ground productivity of terrestrial ecosystems. However, the magnitude and determinants of its effects vary among grassland ecosystems (Jiao et al., 2016; Li et al., 2018; Stan et al., 2020). Our results indicated that the plant biomass in the UN meadow decreased with the change in hydrothermal conditions from 2015 to 2018. In 2015, under the same hydrothermal conditions, the biomass at both the MO and MG sites was higher than that at the UN plot, indicating that moderate disturbance can increase the plant biomass of lowland meadows under adequate moisture conditions. Overall, the interannual variations in plant biomass from 2015 to 2018 were quite different among the sites under the four utilization modes. Biomass at the MO site showed changes that were consistent with the temperature trends. The results of both the Pearson correlation and stepwise regression analyses revealed a significant positive correlation between temperature and biomass at the MO site. The biomass at the MO site was higher than that at the other three sites in all 3 years except 2016, when it was lower than that at the HG site. These results indicate that MO helped to maintain the high biomass of the *C. meyeriana* meadow. This result is consistent with the results of Bernhardt-Römermann et al. (2011) and indicates that moderate mowing can help maintain high grassland productivity. Biomass at the MG site remained stable over the 3 years from 2016 to 2018 with no significant differences among years, and it was consistently higher than that at the UN site. These results indicate that moderate grazing helps maintain stable productivity in lowland meadows and are in line with the findings of Zhang et al. (2018c) and Nunes et al. (2019) that moderate grazing or reduced grazing intensity can maintain stable, high grassland productivity.

Biomass at the HG site decreased significantly after 2016 and was lower than that at the UN site in 2017, when the precipitation was much lower than the historical average. However, the structural equation model indicated that in this experiment, the increase in grazing intensity helped increase the biomass of the lowland meadow. The reasons for this contradiction may be that the soil moisture in the lowland meadow was higher under natural conditions than under grazed conditions and that grassland vegetation is typically water-loving and not drought-tolerant. The results in 2017 showed that it is a combination of factors such as climate and grazing that determine grassland plant biomass. In dry years, plants cannot grow normally, and precipitation becomes a stronger limiting factor than overgrazing for plant growth (Liu, 2019). Furthermore, Bai et al. (2012) suggested that heavy grazing accelerates the impacts of climate change on grassland ecosystems. However, Irisarri et al. (2016) found that the impacts of precipitation on semi-arid dwarf grasslands and mixed grasslands in North America were not affected by grazing; this divergent result may be related to the study differences in the site conditions of grasslands.

## Conclusion

Under global climate change in a *Carex meyeriana* lowland meadows, MO was identified as the most appropriate utilization mode for maintaining the overall stability of the plant community and the soil physicochemical properties, and MG was the best utilization mode for maintaining higher plant community stability and plant biomass. In addition, the MG site was the site most similar to the original grassland in its plant and soil physicochemical properties. Within a certain range, an increase in grazing intensity helped increase plant biomass in *C. meyeriana* lowland meadows. Under grazed conditions, an increase in temperature also increased plant biomass in *C. meyeriana* lowland meadow.

## Data Availability Statement

The original contributions presented in the study are included in the article/Supplementary Material, further inquiries can be directed to the corresponding author/s.

## Author Contributions

GJ and BL were responsible for the data analysis and article writing. HY and GL assisted in the field test. YY was responsible for editing the manuscript. GC provided the experimental ideas, experimental guidance, and financial support. All authors contributed to the article and approved the submitted version.

## Conflict of Interest

The authors declare that the research was conducted in the absence of any commercial or financial relationships that could be construed as a potential conflict of interest.
