# Supplementary material for: Non-utilization Is Not the Best Way to Manage Lowland Meadows in Hulun Buir
Source: Front Plant Sci. 2021 Jul 16;12:704511. doi: 10.3389/fpls.2021.704511 (PMC8322850; doi:10.3389/fpls.2021.704511)
Supplement: Supplementary file 1 [file Data_Sheet_1.docx]

Table 1

Fitting function of grassland plant community stability under different utilization modes

| Year | Plot | Fitting function | R^2^ | Cross point (x, y) | Distance |
| --- | --- | --- | --- | --- | --- |
| 2015 | UN | y = -0.0005x^2^ + 0.4518x + 0.6483 | 0.9761 | (70.13, 29.87) | 70.89 |
|  | MO | y = -0.0007x^2^ + 0.4681x + 0.5296 | 0.9788 | (70.10, 29.90) | 70.85 |
|  | MG | y = -0.0009x^2^ + 0.4857x + 0.3832 | 0.9820 | (70.02, 29.26) | 70.74 |
|  | HG | y = -0.0009x^2^ + 0.4847x + 0.3358 | 0.9924 | (70.11, 29.89) | 70.86 |
| 2016 | UN | y = -0.0004x^2^ + 0.4439x + 0.6989 | 0.9966 | (70.14, 29.86) | 70.90 |
|  | MO | y = -0.0005x^2^ + 0.4531x + 0.6295 | 0.9938 | (70.07, 29.93) | 70.82 |
|  | MG | y = -0.0008x^2^ + 0.4779x + 0.4077 | 0.9791 | (70.04, 29.96) | 70.77 |
|  | HG | y = -0.0007x^2^ + 0.4702x + 0.4559 | 0.9848 | (70.04, 29.96) | 70.77 |
| 2017 | UN | y = -0.0008x^2^ + 0.4749x + 0.4955 | 0.9896 | (70.13, 29.87) | 70.90 |
|  | MO | y = -0.0005x^2^ + 0.4557x + 0.5376 | 0.9839 | (70.01, 29.99) | 70.72 |
|  | MG | y = -0.0007x^2^ + 0.4701x + 0.4674 | 0.9836 | (70.04, 29.96) | 70.77 |
|  | HG | y = -0.0001x^2^ + 0.4309x + 0.3249 | 0.9752 | (70.00, 30.00) | 70.71 |
| 2018 | UN | y = -0.0008x^2^ + 0.4757x + 0.4520 | 0.9925 | (70.12, 29.88) | 70.89 |
|  | MO | y = -0.0005x^2^ + 0.4537x + 0.6038 | 0.9802 | (70.06, 29.94) | 70.80 |
|  | MG | y = -0.0001x^2^ + 0.4293x + 0.3467 | 0.9716 | (70.07, 29.93) | 70.80 |
|  | HG | y = -0.0006x^2^ + 0.4623x + 0.5050 | 0.9903 | (70.05, 29.95) | 70.79 |

Table 2 Plant dominance from 2015-2018

| Plot | Classification | 2015 | 2016 | 2017 | 2018 |
| --- | --- | --- | --- | --- | --- |
| UN | *Carex meyeriana* | 0.7068±0.090 | 0.7863±0.044 | 0.5009±0.081 | 0.6246±0.105 |
|  | Other grasses | 0.2932±0.056 | 0.2137±0.015 | 0.4991±0.094 | 0.3754±0.025 |
| MO | *Carex meyeriana* | 0.3228±0.025 | 0.3752±0.084 | 0.4575±0.053 | 0.2856±0.046 |
|  | Other grasses | 0.6772±0.006 | 0.6248±0.050 | 0.5424±0.105 | 0.7144±0.094 |
| MG | *Carex meyeriana* | 0.4772±0.080 | 0.6843±0.105 | 0.5623±0.115 | 0.6069±0.104 |
|  | Other grasses | 0.5227±0.058 | 0.3157±0.032 | 0.4378±0.060 | 0.3931±0.055 |
| HG | *Carex meyeriana* | 0.3246±0.090 | 0.273±0.068 | 0.228±0.034 | 0.5595±0.064 |
|  | Other grasses | 0.6754±0.084 | 0.727±0.050 | 0.7719±0.060 | 0.4405±0.057 |

Table 3a Correlation analysis of plant community characteristics and soil physicochemical properties in lowland meadow

| Plot | Indicators | Soil layer  （cm） | PB | Richness | Shannon-Weiner | Simpson | Pielou |
| --- | --- | --- | --- | --- | --- | --- | --- |
| UN | SBD | 0～20 | -0.6776 | -0.8545 | -0.8075 | -0.9861* | -0.5617 |
|  |  | 21～40 | -0.5365 | -0.8181 | -0.7520 | -0.9605* | -0.6188 |
|  | SM | 0～20 | 0.6214 | 0.1261 | 0.2480 | -0.0515 | -0.2467 |
|  |  | 21～40 | 0.5675 | 0.1466 | 0.2695 | -0.0759 | -0.1597 |
|  | pH | 0～20 | 0.7276 | 0.8582 | 0.9107 | 0.6338 | 0.6672 |
|  |  | 21～40 | 0.4694 | 0.1732 | 0.2930 | -0.1073 | -0.0248 |
|  | SOM | 0～20 | -0.1652 | -0.8055 | -0.7545 | -0.6839 | -0.995** |
|  |  | 21～40 | -0.6740 | 0.0319 | -0.0488 | -0.0602 | 0.6046 |
|  | STN | 0～20 | -0.3669 | -0.8961 | -0.8373 | -0.8737 | -0.9201 |
|  |  | 21～40 | 0.4819 | 0.8245 | 0.8562 | 0.5564 | 0.8310 |
|  | AN | 0～20 | 0.1804 | -0.3437 | -0.3573 | -0.0120 | -0.7302 |
|  |  | 21～40 | -0.8952 | -0.3612 | -0.4521 | -0.3395 | 0.2073 |
|  | AP | 0～20 | 0.9999** | 0.7248 | 0.7766 | 0.7275 | 0.1874 |
|  |  | 21～40 | -0.9090 | -0.4301 | -0.5236 | -0.3713 | 0.1114 |
|  | AK | 0～20 | -0.7691 | -0.1496 | -0.2531 | -0.1153 | 0.3868 |
|  |  | 21～40 | -0.8353 | -0.7791 | -0.8515 | -0.5801 | -0.4675 |
| MO | SBD | 0～20 | -0.0853 | 0.7968 | 0.0233 | -0.0370 | -0.0138 |
|  |  | 21～40 | 0.4954 | 0.6350 | -0.5015 | -0.5582 | -0.7679 |
|  | SM | 0～20 | 0.0483 | 0.1399 | 0.9541* | 0.9587* | 0.9875* |
|  |  | 21～40 | 0.0610 | 0.1159 | 0.9590* | 0.9656* | 0.9903** |
|  | pH | 0～20 | -0.1672 | -0.5253 | 0.6921 | 0.7433 | 0.8436 |
|  |  | 21～40 | -0.6172 | -0.8603 | 0.1874 | 0.2562 | 0.4965 |
|  | SOM | 0～20 | -0.2539 | -0.9407** | -0.3167 | -0.2500 | -0.1644 |
|  |  | 21～40 | -0.5695 | -0.4758 | -0.9557** | -0.9345** | -0.773** |
|  | STN | 0～20 | -0.5178 | -0.8870** | -0.6602 | -0.6030 | -0.4404 |
|  |  | 21～40 | 0.5744 | -0.0304 | -0.5042 | -0.5103 | -0.7413 |
|  | AN | 0～20 | -0.4265 | -0.8214 | 0.3367 | 0.4048 | 0.5837 |
|  |  | 21～40 | -0.3516 | -0.2601 | -0.9999** | -0.9960** | -0.917** |
|  | AP | 0～20 | 0.3962 | 0.3228 | 0.9964** | 0.9877* | 0.8917** |
|  |  | 21～40 | 0.5283 | 0.9993** | 0.2099 | 0.1369 | -0.0532 |
|  | AK | 0～20 | -0.7720 | -0.3113 | 0.3311 | 0.3605 | 0.6576 |
|  |  | 21～40 | -0.5123 | -0.6592 | 0.4734 | 0.5315 | 0.7462 |

Table 3b Correlation analysis of plant community and soil physicochemical properties in lowland meadow

| Plot | Indicators | Soil layer  （cm） | PB | Richness | Shannon-Weiner | Simpson | Pielou |
| --- | --- | --- | --- | --- | --- | --- | --- |
| MG | SBD | 0～20 | -0.6294 | -0.5332 | -0.4577 | -0.3854 | -0.3795 |
|  |  | 21～40 | -0.9736* | -0.9669* | -0.9994** | -0.9790* | -0.9847* |
|  | SM | 0～20 | 0.7357 | 0.7046 | 0.5584 | 0.4394 | 0.4473 |
|  |  | 21～40 | 0.7254 | 0.7120 | 0.5461 | 0.4157 | 0.4270 |
|  | pH | 0～20 | 0.9995** | 0.9821* | 0.9648** | 0.9131 | 0.9196 |
|  |  | 21～40 | 0.9911** | 0.9749* | 0.9949** | 0.9661* | 0.9711* |
|  | SOM | 0～20 | -0.9178 | -0.8583 | -0.8138 | -0.7486 | -0.7493 |
|  |  | 21～40 | -0.9885* | -0.9478 | -0.9400 | -0.8943 | -0.8968 |
|  | STN | 0～20 | 0.9588 | 0.9281 | 0.9942* | 0.9946* | 0.9951* |
|  |  | 21～40 | -0.9626* | -0.9205 | -0.8793 | -0.8144 | -0.8179 |
|  | AN | 0～20 | -0.8716 | -0.7946 | -0.7578 | -0.6992 | -0.6965 |
|  |  | 21～40 | -0.6983 | -0.5870 | -0.5581 | -0.5111 | -0.5015 |
|  | AP | 0～20 | 0.6485 | 0.5308 | 0.5033 | 0.4591 | 0.4480 |
|  |  | 21～40 | 0.3781 | 0.2543 | 0.1982 | 0.1477 | 0.1347 |
|  | AK | 0～20 | 0.9052 | 0.8369 | 0.8013 | 0.7420 | 0.7408 |
|  |  | 21～40 | 0.6133 | 0.5806 | 0.4133 | 0.2864 | 0.2940 |
| HG | SBD | 0～20 | -0.8730 | -0.9309** | -0.9046 | -0.7200 | -0.7632 |
|  |  | 21～40 | -0.8790 | -0.9753** | -0.9409 | -0.8320 | -0.8510 |
|  | SM | 0～20 | 0.8011** | 0.6326 | 0.3660 | 0.0524 | 0.1101 |
|  |  | 21～40 | 0.7047 | 0.6369 | 0.5850 | 0.2615 | 0.3467 |
|  | pH | 0～20 | 0.9088 | 0.9814* | 0.9155 | 0.7865 | 0.8079 |
|  |  | 21～40 | 0.2213 | 0.4341 | 0.8220 | 0.7162 | 0.7880 |
|  | SOM | 0～20 | -0.4313 | -0.6406 | -0.9394** | -0.8454** | -0.901** |
|  |  | 21～40 | -0.6739 | -0.7583** | -0.8811 | -0.6591 | -0.7324 |
|  | STN | 0～20 | 0.9818* | 0.9026 | 0.5439 | 0.4233 | 0.4095 |
|  |  | 21～40 | -0.6213 | -0.6800 | -0.8082 | -0.5532 | -0.6379 |
|  | AN | 0～20 | -0.3115 | -0.5048 | -0.8524 | -0.7214 | -0.7955 |
|  |  | 21～40 | -0.9997** | -0.9526* | -0.6792 | -0.5201 | -0.5271 |
|  | AP | 0～20 | -0.9966** | -0.9575* | -0.7084 | -0.5392 | -0.5521 |
|  |  | 21～40 | -0.9058 | -0.8069 | -0.3814 | -0.3236 | -0.2842 |
|  | AK | 0～20 | -0.7918 | -0.8744 | -0.9245 | -0.7332 | -0.7877 |
|  |  | 21～40 | -0.9127 | -0.8228 | -0.4079 | -0.3525 | -0.3134 |
